# The coping strategies of front-line health workers in the context of user fee exemptions in Niger

**DOI:** 10.1186/1472-6963-15-S3-S1

**Published:** 2015-11-06

**Authors:** Aïssa Diarra, Abdoulaye Ousseini

**Affiliations:** 1 Laboratoire d'Etudes et de Recherche sur les Dynamiques Sociales et le Développement Local (LASDEL), BP 12901, Niamey, Niger

## Abstract

When user fee exemptions were introduced for children under five years of age in Niger, front-line staff in the health system were not consulted in advance, and various obstacles seriously hindered the policy's implementation. Health workers developed two types of coping strategies. The first dealt with shortcomings of the policy implementation process related to management tools, drug stocks, co-existence of the fee exemption and cost recovery systems, and, above all, supply management for medicines (ordering from private companies, issuing makeshift prescriptions). The second involved clientelism, circumvention of regulations, and misappropriation of resources. Adverse effects have arisen due to both the failings of the health system and the practices of health workers. These include a focus on the commercial management of patients, the most 'costly' of whom sometimes find themselves being refused treatment, patients roaming in search of medicines and treatment, and a decline in quality of care.

## Introduction

The increase in the number of children under the age of five attending health facilities over the past seven years in Niger is unprecedented. Whereas in 2005 there were 1,253,199 new consultations recorded for children under five, in 2010, that number reached 5,554,288 [1,2]. This sudden increase in the use of health services by this age group has been largely linked to recent user fee exemption policies [3], although other strategies have, in the past, had similar but less spectacular effects. These have included the construction of new health centres and the creation of health districts under the Bamako Initiative, large-scale vaccination campaigns, the Integrated Management of Childhood Illness (IMCI) program, and the childhood malnutrition eradication program. In April 2006, the Nigerien State introduced full fee exemption for preventive and curative healthcare for children under five. This targeted abolition marked the end of point-of-service user fees for this age group, although fees still applied for the majority of paediatric services. It should be noted, also, that some services were already free, in particular those of a preventive nature, due more to certain policies that predated the Bamako Initiative than to vertical programs such as vaccinations.

It was against an international backdrop of public health considerations combined with the pursuit of Millennium Development Goals (MDG) [4,5] that Niger, like other West African countries, introduced this new fee exemption policy [6]. The same spectacular increase in curative paediatric consultations has been observed in all countries where such policies have been introduced. However, undesirable effects have also manifested, such as declining quality of care (mainly due to drug shortages), healthcare workers' perceptions of increased workloads, and weakening of health centres' financial positions caused by reimbursement delays. These impacts have already been examined in several studies [7-11]. However, those studies are inadequate in several ways: i) they rely essentially on quantitative methods, and those that include qualitative data do so only very partially; ii) they tend to highlight service use increases without sufficiently analysing implementation; and iii) no qualitative study has focused specifically on the implementation of fee exemption measures for children in health centres. A 2009 review of the literature highlighted some of these research gaps [12] and, to our knowledge, these persist in more recent publications. In West Africa, in particular, health anthropology has shown only a marginal interest in the recent fee exemption measures compared to other disciplines, such as public health and epidemiology. In this article we hope to narrow this gap somewhat by deciphering the coping strategies adopted by health workers when implementing fee exemptions--strategies that include practices, interactions, negotiations, and contradictions. By using a qualitative approach, our aim is to give substance to the quantitative data [13] in order to better understand the successes and failures of the implementation of the user fee exemption for children under five.

### Method

The approach we adopted involved conducting monographic studies in which we observed actors and listened to their statements, in four health districts (DS) of Niger: *Gaweye*, in the urban community of Niamey; *Say*, a rural to semi-rural district 54 km outside Niamey in Tillabéry region; and *Dosso *and *Loga*, two DSs in Dosso region, 136 km from Niamey. The studies were carried out over different periods between 2009 and 2011, with surveys lasting an average of two months per site [14,15].

The semi-structured interviews were conducted with men and women from eight strategic groups: (1) administrative personnel; (2) healthcare delivery teams at all levels; (3) support staff (e.g. unskilled workers, ambulance drivers); (4) management committee (COGES) members; (5) paying users; (6) parents of children eligible for the fee exemption; (7) non-governmental organizations (NGOs); and (8) private pharmacies. We met them either in the healthcare facilities or, for users, in their homes. We carried out a total of 142 formal and informal interviews, 12 of which were group interviews. Altogether, 177 people were interviewed.

**Table 1:** Interviews per strategic group

Strategic groups	Respondents	Individual interviews	Group interviews	Total
*Administrative personnel*	Regional health authority (DRSP) Regional hospital (CHR) District supervisory team (ECD)	11		11

*Healthcare delivery teams*	Physicians Nurses Pharmacy managers Fee collectors	57	3	60

*COGES members *		26	4	30

*Support personnel*	Unskilled workers, ambulance drivers	13		13

*NGO*		1		1

*Private pharmacy*		1		1

*Paying users*		3	3	6

*Fee exemption beneficiaries*	Parents of children under 5 years of age	18	2	20

**Total**		**130**	**12**	**142**

We consulted a wide range of documents: forms and checklists for supervision, stock control, and identification of indigents; purchase orders for materials, consumables, and essential generic drugs (EGDs); treatment guidelines; notebooks; receipts; discharge registers; prescription registers; fee exemption registers; and EGD distribution registers. Observations were carried out in the four health districts covered by the study, in particular in the four district hospitals and five integrated health centres. The collected data were processed using thematic analysis.

### Context

After six years of implementation of the user fee exemption for children under five, Niger remains one of the countries with the worst health indicators in the world for this age group. Despite having decreased from 273.8/1,000 in 1998 to 130.5/1,000 in 2010, infant and child mortality remains at a critical level [16]. Many children continue to die of malaria, diarrhoeal diseases, respiratory disorders, meningitis, and measles in the first five years of life. Health coverage in 2010 was estimated at 49.44% [2]. Health infrastructure and equipment are inadequate and frequently defective. There are serious human resources problems: shortages, poor staff allocation, and excessive health worker mobility.

The decree ratifying user fee exemptions for children under five was issued in April 2006. The recent fee exemption measures were essentially financed using the State's own resources, but with temporary financial support from the Agence Française de Développement (AFD), assistance in the form of inputs from international institutions such as the Global Fund and UNICEF, and local support from some NGOs. Health facilities are reimbursed retroactively according to fixed prices assigned to each of the free services provided, which are based on the cost recovery pricing system introduced in the early 1990s. Theoretically, the free services provided over the course of one month are supposed to be reimbursed at the end of the following month. The DS, which was our primary level of investigation within the health pyramid, generally comprises a district hospital (HD), some integrated health centres (CSI), and, at the front-line, dispensaries (CS). At the time of our study, three of our four DSs, i.e., Gaweye, Say, and Dosso, were not receiving any community-based support from international NGOs; the fourth, Loga DS, was supported by Mercy Corps.

Like the entire country, the four DSs operate in accordance with Bamako Initiative principles. Nonetheless, they all participate in the same common fund; that is, the cost-recovery revenues generated by healthcare facilities are centralized at the district level. This joint fund is managed by the departmental management committee (i.e., the district COGES) and the district management team (ECD). It is used to purchase EGDs and management tools, and to pay the salaries of certain contractual workers as well as fixed costs, such as water and electricity bills.

### Initial trials and errors

In the four DSs under study, as in the rest of the country, the user fee exemption was officially introduced, in most cases verbally, to health workers at general meetings held in the districts. In Dosso region the announcement was made within a month after the decree. In Say and Gaweye, on the other hand, these general meetings were held later, in 2007. The offhand way in which the announcement was made in Gaweye, in particular, is noteworthy: at a general meeting to present the results of health facility audits, information about the fee exemption was a 'miscellaneous' agenda item, relegated to the end of the meeting.

The health workers received the news of the new system with considerable concern:

*"Who will reimburse all the money the hospitals will spend on medicines, or on management tools and other things?!" *(Health worker, Gaweye DS)

*"I asked whether we're going to deliver children's products, or what?" *(District COGES member, Say DS)

*"We told him *[the Regional Director of Public Health] *all our concerns regarding the application of this fee exemption and the risks for us in terms of drug stock-outs, and especially *[regarding the fact] *that nothing had been done or planned prior to the adoption of this fee exemption system." *(Manager, Loga DS)

Based on the imperative tone used by the health authorities, all the health workers perceived the announcement as an order. Three expressions surfaced repeatedly in their descriptions of it: "an order", "a directive", "this is not open to discussion".

Many members of the health committees (COSA) and management committees (COGES) in the different healthcare facilities did not agree with the measure, but they implemented it. The COGESs of Loga and Dosso considered the fee exemption to be an attack on community participation. They saw it as something imposed by the government and believed they had no option but to submit to it.

In reality, the main bearers of the information in the DS did not understand the nature of this new measure themselves. Their task was rendered all the more difficult by the fact that they were not equipped with arguments for "getting the message across". They were merely the conveyors of information from above, i.e., from the Office of the President and the Ministry of Public Health, and had played no part in formulating either the measure or the conditions of its implementation. When faced with the health workers' urgent questions regarding how this measure would actually be implemented on the ground, the managers were evasive. As one health worker told us:

*"What are they going to answer? They said this was just a beginning, they would see, they themselves couldn't say anything about it. They said it was a system they wanted to establish and that they would see later." *(Health worker, Gaweye DS)

Thus the information sessions in the districts were dominated by a feeling of uncertainty and a sense of embarking on new ground. The uneasiness of certain members of the ECD should be pointed out here, in particular the communicators (the most senior people), who had long defended and carried the message of the Bamako Initiative to the public and now had to convey to those same people the opposite message of fee exemption. In other words, it was difficult for them to have to defend the requirement for financial contributions from the community, on one hand, while also talking about exempting over half of the health centres' patients, on the other. As one management team member in a district (Filingué) not included in this study lamented: "*If you take a decision, you have to take responsibility for it. I passed on the information about cost recovery, and now it *[healthcare] *is free of charge...."*

This perceived contradiction in the change of direction of health policy generated a lot of frustration among the health workers, who had been convinced of the rightness of the earlier measures.

When providing free consultations and medicines to the beneficiaries, the health workers had to write receipts to obtain reimbursement from the State. In reality, nobody knew what needed to be done. In the beginning, the receipts were carefully stored in boxes and presented together with summary sheets, which served as invoices, to the fee exemption unit at the end of the month. The latter was soon swamped by a deluge of receipts from all the DSs and requested that only summary invoices be submitted, without receipts. Adjusting to the new system also affected the stocking of drugs, which involved physically separating those intended for the cost recovery system, that is, for the resale pharmacy, from those reserved for distribution under the fee exemption system. The operating mechanism was slightly modified in certain districts that had opted for the 'segregated' cost recovery approach, in which services were charged separately from drugs. This approach was replaced everywhere by the fixed-price approach (covering both services and drugs), as the fee exemption system is based on fixed-price reimbursement. Lastly, functions such as the 'fee-exemption focal point' were created within the regional health authorities (DRSP) and district management teams (ECD) to ensure transparency in the fee exemption system monitoring and management.

The abrupt introduction of the fee exemption policy reflected an enormous lack of preparation. It was also deeply indicative of the gulf between a central level where all decision-making and development of technical tools are concentrated and a peripheral level that is relegated to a purely implementation role.

### Ups and downs in the user fee exemption implementation

Operational deficiencies became apparent from the outset and persisted. Yet certain districts overcame some of the difficulties and managed to implement the system better than others. In this section we specifically examine, for each of these two scenarios, the coping strategies adopted by the health workers and users to overcome the constraints of the system, as well as their outcomes.

#### Stocking essential generic drugs on credit

The National Office for Pharmaceutical and Chemical Products (ONPPC) is the central public agency responsible for importing medical inputs, in particular EGDs, and supplying them to the health centres at affordable prices. In principle, the health centres are supposed to submit their orders for inputs to the State enterprise. If a district's requirement cannot be met there, it then can approach private companies. However, the ONPPC has been experiencing management difficulties for some time and has been unable to fulfil its role as main supplier due to financial and organisational crises that cause frequent breakdowns in supplies [17]. These breakdowns can involve not only much-needed drugs, but also more common products that it is difficult to imagine running out of in a healthcare facility, such as "simple cotton wool", as one health worker reported.

Moreover, a large part of the State subsidies paid to health facilities goes through the ONPPC in the form of drug allocations. However, due to the latter's management problems, various DSs do not manage to obtain these EGDs. Conversely, sometimes the DSs receive supplies of drugs they already have in abundance.

*"For example, they say: 'you ordered 200, we're giving you 50.' For the cost of the other 150, you have to see what's available from them, even if nothing else is needed. You have to take what they have in bulk to fulfil the order." *(Health worker, Say DS)

Thus the health centres are not supplied on the basis of their orders, but rather according to what products are available from the ONPPC. These problems are compounded by the long wait for deliveries. For these reasons, most health centres have gradually ended up using private companies as their main suppliers, even though these are only meant to have a complementary role to the ONPPC. Health centres enjoy more flexible terms with private companies, an important consideration given their reduced income: they pay less for certain products than with the ONPPC (the trade-off being potentially poorer quality and sub-optimal storage), supplies can be purchased on credit, and products are delivered quickly. Moreover, they qualify for discounts, generally referred to as 'rebates', which vary between five and ten percent of order totals. In some instances, these 'rebates' are actually hidden commissions pocketed by the person submitting the order and not the health centre. Thus, even while relying on these 'guardian angel suppliers', health workers say they are conscious of the fact that these are, first and foremost, commercial concerns to which the health centres will have to repay enormous debts.

#### Diversity of situations at the district level

If the State had actually managed to reimburse the health centres for the free healthcare provided, those centres' incomes would have doubled or even tripled [18], far surpassing the already considerable income brought in through cost recovery. For example, in 2009 the direct revenue collected between January and April alone in Gaweye DS totalled almost XOF 24,272,350. However, because of erratic reimbursement--the core problem associated with the fee exemption policy--COGESs have been forced to deplete any financial reserves they had managed to accumulate.

This financial failure was not consistent across the country, however, and there are a few rare DSs that have fared better financially. One such case is Loga DS. Like all the health centres, it experienced difficulties in the early days of the fee exemption, but unlike many others, those problems disappeared with the first reimbursements in 2008. In 2010, the district had XOF 48,000,000 in non-reimbursed invoices, but it had a bank balance of XOF 35,000,000 and drug stocks with an estimated value of XOF 30,000,000. Because of this, the district health centre of Loga was identified as a 'model' in its local governance of the fee exemption, and in August 2010 a mission from the COGES of Aguié DS went there to learn from its experience. Its success is ascribed to rigorous organisation orchestrated by the ECD, which ensures regular processing of data and careful monitoring of management practices in the healthcare facilities. These good management practices enabled the district to submit its invoices promptly. Moreover, as mentioned above, the district benefits from the local support of the NGO Mercy Corps, which donated drugs worth XOF 69,000,000 in 2009. The NGO also provided training in emergency obstetric care and in management and community-building for the members of the COSA and managers of the healthcare facilities. This support helped the district to avoid EDG stock-outs and provided access to sufficient funds to ensure its operations. We have analysed similar support received in other districts of Niger, such as Keita, assisted by Médecins du Monde [19] and Téra and Mayahi, assisted by Help (Ridde, Diarra, Moha, unpublished report, available from author). Nevertheless, good availability of financing and of EGDs does not necessarily translate into a high level of attendance. One study showed that the number of visits recorded in Loga DS (before and since the fee exemption) was limited in comparison to other districts (Baroy and Laouali, unpublished report, available from author). This may partially explain its coping ability, if we assume that fewer visits also led to fewer problems than in other districts with higher attendance.

These variations between the districts implicitly confirm that measures for removing financial barriers are not always sufficient to ensure strong attendance at healthcare services. Factors associated with the healthcare system itself (problems with the referral/evacuation system, quality of care, clientelism, favouritism, poor management, etc.) can also, like sociocultural and religious factors, present obstacles to accessing and using health services [20]. These pose a real threat to the sustainability of health policies.

#### Governing the implementation of fee exemption with the legacies of the past

The 'directive-to-implementation' logic referred to above--typical of the health system and in play from the outset of the fee exemption, and which contributed to the lack of preparation described earlier--continued to dominate with the 'extra centimes' affair. In effect, while caesareans were free of charge, patients still had to pay the evacuation costs, which could equal or exceed the cost of the procedure itself. To cope with this situation, a payment of XOF 100 was exacted from each patient at every episode of ill health and put toward funding free medical evacuations [21]. This initiative originated in a district of the Dosso region and spread throughout Niger. While it proved to be effective in providing free evacuations, the Ministry of Public Health swiftly prohibited the inclusion of fee exemption beneficiaries in this system. According to the health authorities, in making those beneficiaries pay this extra contribution, health centres were contravening the fee exemption policy. This decision, which did not propose any alternative strategy, struck a serious blow to the referral/evacuation system, which relied heavily on these 'extra centimes'. Some districts were compelled to stop the free evacuations, even though problems associated with medical evacuations are known to be a contributing factor in the lack of continuity of care for fee exemption beneficiaries.

Another directive was issued during the implementation: a ban on issuing prescriptions to patients targeted by the fee exemption. Health workers were expected to draw from the funds generated by cost recovery to provide free treatment to patients, pending reimbursement. However, those reserves were depleted due to very long reimbursement delays [22], which in turn impeded the replenishment of EGD stocks. Because of stock-outs, health workers were unable to provide free medicines to the patients and so issued them with prescriptions. The State prohibited this, thereby obliging them to send patients away not only with no drugs, but also with no possibility of buying them at the pharmacy. When faced with this dilemma, some health workers followed the directive despite their own misgivings, whereas others defied it, either by providing anonymous prescriptions on simple scraps of paper or by issuing properly completed prescription forms. They justified their actions primarily on the basis of ethical considerations:

*"You cannot turn a child away. We have a responsibility here." *(Medical officer, Gaweye HD)

*"A child comes and we have no drugs and don't issue a prescription. We feel sorry for them. I, myself, issue the prescription." *(Nurse, Gaweye CSI)

In reality, these problems that surfaced in the interactions between the central and peripheral levels reflect a bureaucratic governance model that is extremely hierarchical [23]. Drug stock-out problems also occurred in Ghana, prompting a return to a user payment system in certain districts there [24].

Other structural malfunctions of the healthcare system manifested in key services across all levels of the hierarchy. We observed them particularly in the management of medicines. The issue of the unavailability of drugs is not only a financial one, but very likely also one of management. For instance, when stocks of paediatric syrup were used up, even though paediatric tablets also exist, these were not provided to the patients. Various orders were delayed because of managers' negligence. Sometimes managers were unaware of their centre's average monthly consumption levels, with the result that purchase orders were not updated to reflect real increases in needs. On the other hand, we observed that certain malfunctions were not due to incompetence, but rather to a lack of awareness among the professionals. For example, the task of recording fee exemption data at one EGD point of sale was assigned to a national service conscript, but this person was rarely on site, so the forms accumulated and the transmission of data to the upper level was delayed.

Thus supplies are allocated randomly and bear no relation to actual requirements. Urgent purchase orders, which should be exceptional, have become common. There are unexplained losses of products. As one CHR noted, certain practices of health workers result in medicines 'evaporating':

- *Unrecorded removal of medicines*: When the post is very busy, nurses take products for certain patients directly from the sales counter and sometimes fail to return with payment.

- *Invalid requests for payment*: All prescriptions issued under the fee exemption policy must be given a collection number prior to being filled. However, some nurses put false numbers on the orders to procure medicines wrongfully. When these orders are discovered at the administrative level, they become ineligible for reimbursement under the fee exemption system and result in losses for the healthcare facility.

- *Falsified prescriptions for non-exempted patients submitted under the fee exemption account*: This is often done to help a relative avoid paying for drugs, or else to inflate the bill for reimbursement. Parents also lie about their children's ages so as to benefit from the fee exemption. Likewise, very short visits have been known to occur in which no medicines were given out, but for which the State was nevertheless charged. All of this undermines the reliability of the data provided by the healthcare facilities to the statistical data processing services and to the central fee exemption unit.

These malfunctions in the management of medicines point to a problem with control upstream in the system. Despite the existence of mechanisms for the purpose--supervision, physical separation of fee exemption and cost-recovery EGDs, monitoring software (Sage)--there is an almost total lack of control. These mechanisms are either not spoken about or not known, and the attitude towards contraventions is lax. Jaffré explains that *"*everyone belongs to different networks of influence, which discourages complaints" (author's translation) [25]. Very often, contraventions are not punished. Health centres are places where official norms and everyday practices are interwoven and give rise to a "local professional culture" among health workers that is often far removed from regulations and procedures [23]. This impunity is at the root of the widespread absence of strict control or monitoring. These structural malfunctions, the legacy of a health system that has undergone numerous reforms, cannot be rectified with a simple wave of the hand, and they weigh heavily on any new health policy introduced, such as fee exemption.

#### Coping strategies and adverse effects

Faced with the contradictions and shortcomings of the fee exemption policy, health workers have developed a range of coping strategies:

- Some seek out local partners, in particular NGOs, for support in implementing annual programmes of activities; such support may be sporadic or sustained.

- Prescriptions are issued according to product availability. Tablets and capsules in adult doses are given to children (paediatric syrups being far more expensive).

- Referrals between CSIs, from CSIs to the district hospital or, vice versa, from the district hospital to CSIs, have become frequent simply due to the lack of EGDs.

- Prescriptions are given to the beneficiaries of fee exemption, who must then fill them at private pharmacies, where they are required to pay for their medicines.

While these coping strategies may make it possible to continue providing care despite all the problems, they can also have undesired effects on patient management--e.g. patients roaming from one healthcare facility to another in search of treatment; prescriptions not being paid; inappropriate medicines being dispensed whose efficacy is not assured.

As mentioned, the cost recovery system operates through the general application of user fees. According to the health workers, the price charged is much less than the actual cost of treating patients who present with serious complications. These patients consume a lot of products, and the reagents required for their laboratory tests are expensive. Although some informants noted that, conversely, the cost of treating other, less serious illnesses is less than the fee charged, they all subscribe to the view expressed to us by a community worker in Gaweye DS that the fixed-price user fee "has become cheaper for the population and more expensive for the centre!" In some health centres, the strategy used to overcome this perceived shortfall involves selling products to paying patients who did not consult at the healthcare facility. The perverse effects of this situation include the sale of certain medicines (e.g. ciprofloxacin, cloxacillin, penicillin G benzathine injectable suspension, ampicillin) to all patients, whether or not they qualify for exemption, and the classification of users as 'costly', 'very costly' and 'cheap'. Hence, fee exemption beneficiaries, who could be treated in ambulatory care services but are classified as 'costly', are recorded in the register as being 'placed under observation', as this qualifies for higher reimbursement under the fee exemption policy. Moreover, it appears paying patients are less badly treated than those targeted by the fee exemption policy:

*"You arrive at night and knock at the door and they don't even answer, or they tell you to come back, there are no products, particularly when fee exemption is involved. But if you intend to pay, they get up, although with an offended air, unwillingly, and treat you." *(Woman, 35, housewife and mother of five children)

In a context in which users' anonymity exposes them to the risk of maltreatment in the health system [26], this account sounds a warning regarding the emergence of a form of inequality of treatment between paying patients and those who qualify for fee exemption. This commercial logic, which has developed within the health system over the years, has led to extreme situations in which treatment is refused to patients who are exempted from fees. To our knowledge, these perverse effects have not been sufficiently examined, at a time when the international community plans to develop universal health coverage on the African continent [27,28]. It is already known that in the Northern Hemisphere, where many countries have extensive experience with universal health coverage (UHC), the refusal of treatment to beneficiaries of supplementary UHC is one of the persistent bottlenecks in this system [29].

## Conclusion

The difficulties experienced by the State due to the lack of financial and technical resources prior to implementation of the fee exemption policy have aggravated the health system's structural malfunctions. Drug shortages at the service delivery points are the most visible outcome of the financial deficit. This tends to obscure, at the international level, the problems related to the Nigerien State's capacity to supply medicines, manage their flow, and distribute them equitably in all health districts.

Moreover, the policy's success does not depend only on the availability of financing and of EGDs, but also on the performance of the basic healthcare structures, which need to be strengthened to improve quality of care and treatment of complicated cases [30,31]. While health policies are often expected to have positive effects on the provision of care, little consideration is given to the health workers: "Beyond all the talk, they want to see impacts in terms of their own careers, remuneration and development" (authors' translation) [32]. Advocacy, awareness-raising, and debate with these front-line workers were all neglected in the policy implementation process. Any change in the orientation of health policies must necessarily take into account their actual practices, strategies, and capacity to obstruct implementation. In effect, because they are at the heart of the policy implementation process, they also have the power to act positively or negatively on these policies, directly or indirectly [33]. Reforms have a greater chance of success if their formulation and implementation are discussed in detail in advance with the staff. In the cases we studied, such preliminary work would have helped dispel misunderstandings around the seemingly contradictory principles of cost recovery and fee exemption. The pilot experiences were not taken into account in the government's implementation of the fee exemption policy [34]. Lastly, fee exemption is presented as one of the means of achieving the broader objective of universal healthcare coverage [35]. This universal coverage is expected to be achieved through the development of health mutuals, the promotion of health insurance, and the establishment of a social health fund. However, if the internal and external funds required to support this plan are not protected, and if greater attention is not paid to actual front-line care practices and the health system's current malfunctions, there is a risk that it will produce the same negative impacts as those generated by the current fee exemption policy.

## Competing interests

None

## Authors' contributions

AD and AO designed, collected, processed and analysed the data; AD wrote the draft and final version of the manuscript. All authors read and approved the final manuscript.

## Declarations

This article has been modified from the chapter **Les stratégies d'adaptation des agents de première ligne en contexte de gratuité au Niger** by A. Diarra and A. Ousseini in the book *Une politique publique de santé et ses contradictions. La gratuité des soins au Burkina Faso, au Mali et au Niger*, J.-P. Olivier de Sardan and V. Ridde (eds), 2014, Karthala, Paris [36], with the permission of the publisher. The publication of this supplement was funded by a grant from International Development Research Centre (IDRC), Ottawa, Canada.

This article has been published as part of *BMC Health Services *Research Volume 15 Supplement 3, 2015: User Fee Exemption Policies. The full contents of the supplement are available online at http://www.biomedcentral.com/bmchealthservres/supplements/15/S3.
